# Dietary *Dunaliella salina* microalgae enriches eggs with carotenoids and long-chain omega-3 fatty acids, enhancing the antioxidant and immune responses in heat-stressed laying hens

**DOI:** 10.3389/fvets.2025.1545433

**Published:** 2025-02-26

**Authors:** Mahmoud Madkour, Sami I. Ali, Mahmoud Alagawany, Mohamed S. El-Kholy, Farouk K. El-Baz, Abdulmohsen H. Alqhtani, Abdulrahman S. Alharthi, Anthony Pokoo-Aikins, Ahmed A. Elolimy

**Affiliations:** ^1^Animal Production Department, National Research Centre, Giza, Egypt; ^2^Plant Biochemistry Department, National Research Centre, Giza, Egypt; ^3^Poultry Department, Faculty of Agriculture, Zagazig University, Zagazig, Egypt; ^4^Animal Production Department, Food and Agriculture Sciences College, King Saud University, Riyadh, Saudi Arabia; ^5^Toxicology and Mycotoxin Research Unit, U.S. National Poultry Research Center, Agricultural Research Service, U.S. Department of Agriculture, Athens, GA, United States; ^6^Department of Integrative Agriculture, College of Agriculture and Veterinary Medicine, United Arab Emirates University, Al Ain, United Arab Emirates

**Keywords:** *Dunaliella salina*, heat stress, carotenoids, omega-3 fatty acids, laying hens

## Abstract

**Introduction:**

*Dunaliella salina* (DS) is a prospective source of bioactive carotenoids, including beta-carotene, zeaxanthin, and omega-3 fatty acids. The effect of dietary supplementation of DS on the productive performance, immune response, and egg quality of heat-stressed laying hens has not been comprehensively studied. We investigated how dietary *D. salina* supplementation affects the deposition of bioactive carotenoids and omega-3 fatty acids in egg yolks of laying hens experiencing summer heat stress, as determined by the Temperature-Humidity Index (THI). The influence of *D. salina* supplementation on the productive performance, immune response, and antioxidant status of heat-stressed laying hens was assessed.

**Methods:**

A total of 120 Elma-Brown laying hens were assigned to four dietary treatments with DS supplementation at 0 (control), 0.5, 1, and 1.5 g/kg of diet. The experiment lasted 60 days, during which eggs were collected at three time points: 15, 30, and 60 days from the start of the study.

**Results and discussion:**

including DS at 1 g/kg improved egg production and feed conversion, with improved antioxidant status via a marked inhibitory effect on malondialdehyde in heat-stressed laying hens. Dietary 0.5 g/kg DS improved the immune response of heat-stressed laying hens compared to that of the control group. The highest dose of DS (1.5 g/kg diet) increased astaxanthin, zeaxanthin, lutein, and total carotenoids by 9.8%, 50.44%, 49.19%, and 84.21% (*p* < 0.05), respectively, and decreased β-carotene by 38.61% (*p* < 0.05), when compared with the control. Feeding DS to heat-stressed laying hens increased the concentrations of the long-chain Omega-3 (docosahexaenoic acid) in egg yolks; the dose of 0.5 g/kg diet for 15 d produced an increase in the DHA content by104.76% above the control group (*p* < 0.5). Feeding DS improved the nutritional indices of egg yolks, as egg yolks retained a high ratio of monounsaturated fatty acids (MUFA)/polyunsaturated fatty acids (PUFA)/saturated fatty acids, low thrombogenicity index (IT), low atherogenicity index (IA), and high hypocholesterolemic/hypercholesterolemic index (h/H). Feeding heat-stressed laying hens DS improved their productivity and antioxidant status, resulting in functional eggs enriched with bioactive carotenoids (astaxanthin, zeaxanthin, and lutein) and beneficial omega-3 fatty acids.

## 1 Introduction

Climate change is a pressing issue of our time, with unprecedented global consequences. One of the most marked impacts is the alteration of weather patterns, which poses a threat to poultry production (1–9). The severe negative effects of heat stress on chicken productivity and the ensuring sustainability concerns have prompted scientists and poultry producers to seek new and effective solutions (9–12). Numerous natural feed additives have been developed in response to the increasing global demand for antibiotic alternatives in poultry production (13–15). The development of a sustainable strategy to combat heat stress in the poultry industry is imperative. Therefore, developing new feed supplements with functional properties that can mitigate heat stress is critical. To enhance the cellular response to thermal challenges, it is crucial to incorporate antioxidant-rich compounds into the poultry diet to maintain the proper functioning of the redox system (16, 17). The microalgae, *Dunaliella salina* (DS), is rich in omega fatty acids and bioactive carotenoids, including beta-carotene and zeaxanthin (18). This microalgae also contains a wealth of vitamins, minerals, and other bioactive substances such as polyphenolics. The carotenoids in DS, including beta-carotene, lutein, zeaxanthin, and astaxanthin, have potent therapeutic properties (19). Thus, *Dunaliella salina* is a promising feed additive for poultry to improve egg yolk pigmentation, enhance antioxidant status, and boost immune function.

Bioactive carotenoids and omega-3 fatty acids, especially alpha-linolenic acid (ALA), eicosapentaenoic acid (EPA), and docosahexaenoic acid (DHA), found in microalgae, may benefit laying hens by boosting their immune systems and mitigating oxidative stress (20–22). This might mitigate the adverse effects of heat stress in poultry by exerting anti-inflammatory and antioxidant properties. Several studies have demonstrated that the addition of microalgae, including *Nannochloropsis limnetica*, *Nannochloropsis gaditana, Nannochloropsis oculate*, *Porphyridium* sp., *Schizochytrium* sp., and *Chlorella vulgaris* to the diet of poultry has a positive impact on the concentrations of carotenoids and enhanced yolk carotenoids, lutein, B-carotene, and the zeaxanthin concentration (21, 23–25), and fatty acids in laying hens (22, 26).

In the current study, DS was used to enrich the diet of heat-stressed laying hens with carotenoids and omega fatty acids, which served as antioxidant compounds to mitigate the detrimental effects of heat stress. The effects on the nutritional quality of eggs and the performance and physiological responses of laying hens were evaluated. The total carotenoid, beta-carotene, lutein, zeaxanthin, astaxanthin, and fatty acid contents of egg yolks were also assessed.

## 2 Materials and methods

### 2.1 Microalgal biomass

The ground DS biomass used in this study was obtained from the Algal Biotechnology Group, New Central Laboratories Network, at the National Research Centre in Cairo, Egypt.

#### 2.1.1 Preparation of microalgal extract

The fine powder of DS (10 g) was extracted with HIP (hexane:isopropanol, 2:3, v/v) (100 mL, 3 times) for 24 h, centrifuged (Sigma 3-18ks Centrifuge, Germany) at 4,248×*g* for 20 min at 25°C to separate cell debris from the supernatant. The supernatants were evaporated under vacuum (Heidolph Unimax 2010, Germany) at 40°C to obtain the hexane: isopropanol crude extract (HICE). All the extraction steps were performed under dim light (18). HICE was used to determine the carotenoid and fatty acid profiles of DS biomass.

### 2.2 Experimental design and diets

Animal care and maintenance adhered to the guidelines set forth by the Egyptian Research Ethics Committee and Zagazig University for the care and utilization of laboratory animals (approval #ZU-IACUC/2/F/314/2023).

120 Elma-Brown laying hens were used, with three hens in each replicate, following a completely randomized design. The experimental design included four treatments with 10 replicates each. Each replicate was housed in a 50 cm × 50 cm × 45 cm wire pen. The layer house maintained an appropriate lighting schedule (16 h of light and 8 h of darkness) and ensured adequate ventilation conditions. Water and food were provided ad libitum throughout the experiments.

The basal diet was prepared to fulfill the nutrient requirements outlined in the Elma-Brown laying hen management guidelines. Dietary treatments were: (1) control diet (basal diet without DS), (2) basal diet with 0.5 g DS/kg, (3) basal diet with 1 g DS/kg, and (4) basal diet with 1.5 g DS/kg. Experimental diets were provided in mash form, ensuring thorough mixing of DS with all other feed components (27), the formulation and composition of the main diet are shown in Table 1. The duration of the experiment was 60 d (8 weeks) from 45 weeks to 53 weeks of age. The experiment was conducted during summer in Egypt from June 25 to August 23. Ambient temperature and relative humidity were recorded twice daily. The temperature humidity index (THI) elucidates the interplay between temperature and relative humidity, serving as a metric to determine the presence of a heat stress scenario (28):

$$THI={db}^{{}^{\circ}}C-\left[\left(0.31-0.31RH\right)\;\left({db}^{{}^{\circ}}C-14.4\right)\right]\text{.}$$

**Table 1:** Composition and calculated analysis of the basal diets.

Ingredients (%)	
Corn	59.73
Soybean meal, CP 44%	28.02
Soybean oil	1.35
Limestone	8.65
Dicalcium phosphate	1.5
Salt	0.30
Premix^1^	0.30
DL-Methionine	0.15
Calculated composition^2^ (%)	
ME, kcal/kg	2751.6
Crude protein	17.5
Calcium	3.71
Nonphytate P	0.39
TSAA	0.72
Methionine	0.45
Lysine	0.91
Threonine	0.65
Valine	0.81
Crude fiber	3.28
Crude fat	3.76
Linoleic acid	2.16

^1^Vitamin mineral premix contains per kg: Retinyl palmitate, 3.96 mg; vitamin E, 36 mg; vitamin D3, 0.06 mg; riboflavin, 5.6 mg; thiamin mononitrate, 2 mg; niacin (B3), 30 mg; pantothenic acid (B5), 10 mg; folic acid, 0.5 mg, B12, 0.012mg; biotin, 0.22 mg; Mn, 80 mg; Zn, 60 mg; Fe, 80 mg; Cu, 10 mg; I,0.55 mg; Se, 0.12 mg; choline, 250 mg; menadione sodium bisulphite, 4 mg; sodium chloride, 3g.

^2^Calculated according to National Research Council (NRC) 1994.

where db °C = dry bulb temperature (°C) and RH = relative humidity/100. The computed values are then classified as: <27.8 = absence of heat stress, 27.8–28.9 = moderate heat stress, 28.9–30.0 = severe heat stress and > 30.0 = very severe heat stress.

Throughout the study, the average temperature and relative humidity stood at 35.7°C and 63%, respectively (Table 2).

**Table 2:** Temperature-humidity index (THI) throughout the experimental period.

	db °C	RH/100	THI
Week 1	36.20	0.58	33.36
Week 2	32.70	0.59	30.37
Week 3	33.70	0.63	31.49
Week 4	37.60	0.60	34.72
Week 5	36.90	0.61	34.18
Week 6	38.00	0.68	35.66
Week 7	35.60	0.69	33.56
Week 8	35.10	0.66	32.92
Average	35.70	0.63	33.26

### 2.3 Productive performance

All productive performance traits were estimated per replicate on days 15, 30, and 60. Egg production (EP%) and feed intake (FI; g/bird/d) were adjusted for mortality. Egg weight (EW, g) was recorded to the nearest 0.1 g. Egg mass (EM, g/bird/d) was calculated by multiplying EP by EW. The feed conversion ratio (FCR) (g feed/g egg) was calculated as the FI divided by the EM (see Tables 3–5).

**Table 3:** Effect of different levels of *Dunaliella salina* on the productive performance of laying hens at d 15 of the experiment.

Item	Productive performance at d 15				
	EP (%)	EW (g)	EM (g)	FI (g/d)	FCR (g:g)
0	76.2200	59.1500	45.0960	105.7200	2.3540
0.50	88.5800	58.3980	51.7440	105.2400	2.0400
1.00	77.8800	59.5440	46.3740	104.8600	2.2700
1.50	82.4200	58.1640	47.9200	103.9000	2.1760
SEM	1.51052	0.23130	0.85672	0.47783	0.07386
Orthogonal contrast					
Linear	0.446	0.349	0.624	0.204	0.357
Quadratic	0.103	0.466	0.085	0.811	0.145

SEM: Standard Error of Means.

Linear and Quadratic effects.

EP, Egg production; EW, egg weight; EM, egg mass; FI, feed intake; FCR, feed conversion ratio.

**Table 4:** Effect of different levels of *Dunaliella salina* on the productive performance of laying hens at d 30 of the experiment.

Items	Productive performance at d 30				
	EP (%)	EW (g)	EM (g)	FI (g/d)	FCR (g:g)
0	79.1000	60.7400	48.0660	108.8600	2.2780
0.50	83.6600	59.6460	49.8600	107.4200	2.1560
1.00	76.2200	59.9540	45.6920	108.7200	2.3860
1.50	80.3600	59.1560	47.5340	109.0400	2.3000
SEM	1.11186	0.28246	0.65647	0.45900	0.03480
Orthogonal contrast					
Linear	0.691	0.087	0.308	0.670	0.315
Quadratic	0.919	0.789	0.985	0.367	0.781

SEM: Standard Error of Means.

Linear and Quadratic effects.

EP, Egg production; EW, egg weight; EM, egg mass; FI, feed intake; FCR, feed conversion ratio.

**Table 5:** Effect of different levels of *Dunaliella salina* on the productive performance of laying hens at d 60 of the experiment.

Items	Productive performance at d 60				
	EP (%)	EW (g)	EM (g)	FI (g/d)	FCR (g:g)
0	79.7400	59.5020	47.4740	107.8600	2.2840
0.50	79.3200	60.3140	47.8700	107.3200	2.2540
1.00	88.3800	60.2680	53.2760	105.7400	1.9900
1.50	84.8600	59.8120	50.7580	107.0800	2.1140
SEM	1.39960	0.17744	0.90796	0.42822	0.04174
Orthogonal contrast					
Linear	0.037	0.578	0.048	0.317	0.024
Quadratic	0.528	0.088	0.374	0.284	0.284

SEM: Standard Error of Means.

Linear and Quadratic effects.

EP, Egg production; EW, egg weight; EM, egg mass; FI, feed intake; FCR, feed conversion ratio.

#### 2.3.1 Egg quality criteria

Egg quality criteria were examined at the end of the experiment using two eggs from each replicate. Eggs were weighed, and egg length and width were determined before breaking. The eggs were carefully broken on a glass plate (50 × 50 cm) to measure the internal and external quality characteristics. The yolk was separated from the albumen and the eggshell was cleaned of any adhering albumen. The albumen weight was calculated by subtracting the yolk and shell weights from the whole egg weight. The egg shape indices were calculated as the ratio of egg width to egg length (29). Shell thickness was measured using a micrometer (0.01 mm) and reported as the mean value of three measurement regions (air cell, equator, and sharp end). The Haugh unit was calculated as:


$$\mathrm{Haugh unit score}=100\times \log\;\left(H+7.57–1.7EW\times 0.37\right)\text{.}$$

$$\mathrm{Shape index}\%=\frac{\mathrm{Egg}\;\mathrm{width}\;\left(\mathrm{mm}\right)}{\mathrm{Egg}\;\mathrm{length}\;\left(\mathrm{mm}\right)}*100.$$

where H is the albumen height and EW is the egg weight, according to the formula proposed by Eisen et al. (30)

### 2.4 Blood sampling and laboratory analyses

Blood samples were randomly collected from the wing veins of six birds per treatment in sterilized tubes. Samples were left to coagulate and centrifuged at 2,793×*g* for 10 min to obtain serum, which was stored in Eppendorf tubes at −20°C until biochemical analysis (31, 32). The following serum biochemical parameters [total protein (g/dL), albumin (g/dL), globulin fractions (g/dL), AST (U/L), ALT (U/L), creatinine (mg/dL), urea (mg/dL), uric acid (mg/dL), T3 (ng/mL), T4 (ng/mL), glucose (mg/dL), lipid profile parameters (mg/dL), IgG (ng/mL), and IgM (ng/mL)] were estimated in serum using commercial kits using a spectrophotometer (Shimadzu, Japan) according to the procedures described in the pamphlet of kits.

For antioxidant parameters, serum samples were subject to measurement of superoxide dismutase (SOD) activity (U/mL), total antioxidant capacity (TAC, mmol/L), and malondialdehyde (MDA, nmol/mL) using a spectrophotometer (Shimadzu, Japan). SOD activity was measured using the xanthine oxidase method, which monitors the inhibition of nitro blue tetrazolium reduction by the sample (33). The TAC and MDA concentrations were analyzed using a spectrophotometer (34).

### 2.5 Egg collection

Eggs were collected at 15, 30, and 60 d after the start of the experiment. Out of the 10 replicates, eggs (1 from each replicate, 10 per dietary group) were collected and cracked open to separate the yolk. Ten egg yolks from each group were weighed and thoroughly mixed to generate a homogenous sample for carotenoid and fatty acid profile analysis (2 yolks were combined for carotenoid analysis), resulting in 5 samples per treatment each collecting time as reported by Reda et al. (35).

#### 2.5.1 Sample preparation

Two grams of egg yolk and DS were homogenized in a pestle for 5 min using 40 mL of HIP (hexane: isopropanol, 3:2, v/v). To eliminate non-lipids, 25 mL of Na_2_SO_4_ (6.67%) was added (36). The mixture was then poured into a separating funnel, shaken vigorously, and stored until the two layers separated. The upper hexane layer was dried using a rotary evaporator at 40°C and used to analyze carotenoids and fatty acids. All extraction steps were performed under dim light to avoid photooxidation of the carotenoids (37).

### 2.6 Fatty acid analysis

The total lipids in the egg yolk, DS, and diet were methylated according to a method (38). The dried hexane extract (10 mg) of each sample was refluxed with a 30 mL mixture of methanol, benzene, and sulfuric acid (20:10:1, v/v/v) for 2.5 h. The mixture was transferred to a separating funnel, and 60–70 mL of distilled water was added. A small amount of hexane was added, and the upper layer was taken and washed with 50 mL of 10% sodium bicarbonate, and shaken twice. The lower layer was discarded. The upper layer was washed twice with a saturated 0.9% sodium chloride solution. The upper layer was passed through anhydrous sodium sulfate and the extract thus obtained was evaporated. The residue was dissolved in HPLC-grade hexane and analyzed using gas chromatography [GC-flame ionization detector (FID) model 7890B from Agilent Technologies]. Separation was achieved using a Zebron ZB-FAME column (60 m × 0.25 mm internal diameter ×0.25-μm film thickness). Analyses were carried out using hydrogen as the carrier gas at a flow rate of 1.8 mL/min at a split-1:50 mode, injection volume of 1 μL, with the following temperature program: 100°C for 3 min, rising at 2.5°C/min to 240°C, and held for 10 min. The injector and detector were maintained at 250°C and 285°C, respectively.

#### 2.6.1 Fatty acid classification

Fatty acids were classified into saturated fatty acids (SFA), monounsaturated fatty acids (MUFA), and polyunsaturated fatty acids (PUFA):

$$SFA=\left[PA\right]+\left[SA\right]$$

$$\mathrm{MUFA}=\left[OA\right]$$

$$\mathrm{PUFA}=\left[LA\right]+\left[\mathrm{ALA}\right]+\left[DHA\right]$$

where PA = palmitic acid, SA = stearic acid, OA = oleic acid, LA = linoleic acid, and ALA = *α*-linolenic acid.

#### 2.6.2 Lipid nutrition indices of eggs

MUFA/ PUFA and PUFA/ SFA were assessed following the method described by Kurt (39) using the following equations:

$$\mathrm{MUFA}/\mathrm{PUFA}=\frac{\left[OA\right]\;}{\left[LA\right]+\left[\mathrm{ALA}\right]+\left[DHA\right]\;}$$

$$\mathrm{PUFA}/SFA=\frac{\left[LA\right]+\left[\mathrm{ALA}\right]+\left[DHA\right]}{\left[PA\right]+\left[SA\right]\;}$$

The health indices of egg yolks [atherogenicity index (IA), thrombogenicity index (IT), and cholesterolemic index {hypocholesterolemic (h)/hypercholesterolemic index (H) (h/H)}] were determined according to the formula of Wołoszyn et al. (40):

$$IA=\frac{\left[PA\right]+\left[SA\right]}{\left[\mathrm{MUFA}\right]+\left[\mathrm{PUFA}\right]\omega 6+\left[\mathrm{PUFA}\right]\omega 3}$$

$$\mathrm{IT}=\frac{\left[PA\right]+\left[SA\right]}{\begin{array}{l} 0.5x\left[\mathrm{MUFA}\right]+0.5x\left[\mathrm{PUFA}\right]\omega 6+3x\left[\mathrm{PUFA}\right]\omega 3\\ +\frac{\left[\mathrm{PUFA}\right]\omega 3}{\left[\mathrm{PUFA}\right]\omega 6} \end{array}}$$

$$\frac{h}{H}=\frac{\left[OA\right]+\left[LA\right]+\left[\mathrm{ALA}\right]+\left[DHA\right]}{\left[PA\right]\;}$$

### 2.7 Carotenoid analysis

β-carotene, lutein, zeaxanthin, and astaxanthin were identified and quantified using HPLC-diode array detector (DAD) in the hexane extract of egg yolk, DS, and diet, in triplicate, using an Agilent 1260 infinity series HPLC-DAD system (Agilent Technologies, Waldbronn, Germany) equipped with binary gradient Agilent 1260 prep pump (G1361A), and an autosampler Agilent 1260 prep ALS (G2260A). An Agilent diode array detector 1260 DAD VL (G1315D) was used for the detection of the separated β-carotene, lutein, zeaxanthin, and astaxanthin. The separation was performed using an Agilent normal phase (NP) silica column (ZORBAX RX-Sil, 5μm, 4.6 × 150 mm). The following solvents, (A) n-hexane and (B) acetone were used at a flow rate of 1 mL/min using a gradient between solvents A and B, following our previously published method (18): B was run at 0–30% for 5 min, 30–50% for 15 min, 50–100% for 3 min, and maintained at 100% of B until the end of the separation at 30 min. The peaks were integrated at 450 nm to quantify β-carotene, lutein, and zeaxanthin, and at 476 nm to quantify astaxanthin. β-carotene, lutein, zeaxanthin, and astaxanthin (Sigma-Aldrich Co., United States) were used as standards. β-carotene, lutein, zeaxanthin, and astaxanthin were identified and quantified in the extracts by comparing retention time and the peak area of the unknown peak with the standards. The total carotenoid content was quantified using the total peak area relative to the peak area of the β-carotene standard.

### 2.8 Statistical analysis

The experiment was carried out as a completely randomized design. The performance, blood parameter data were evaluated using the GLM procedure of SAS (64). Orthogonal polynomial contrasts were used to test the linear and quadratic effects of the increasing levels of supplementation of DS. Carotenoid content in egg yolk (β-carotene, lutein, zeaxanthin, and astaxanthin) was determined using a two-way analysis of variance using DS levels and collecting period as fixed effects and their interactions using GLM procedure of SAS (64). Tukey’s test at *p* < 0.05 tested differences within means of treatments.

## 3 Results

### 3.1 Laying performance

The inclusion of DS in laying hen diets did not significantly affect egg production (EP), egg mass (EM), egg weight (EW), feed intake (FI), and feed conversion ratio (FCR), at 15 and 30 days of the experiment (as shown in Tables 3, 4), however, *Dunaliella salina* supplementation improved EP and FCR with the best level being 1 g/kg feed compared to the other levels and the control group (linear, *p* < 0.05) (Table 5) at 60 days of the experiment. The addition of DS to laying hen diets did not affect EM, EW, and FI. Inclusion of DS into laying hen diets at 1.5 g/kg increased the Haugh unit (linear, *p* = 0.009; quadratic, *p* = 0.002), with no changes in the other egg quality parameters [shell%, yolk%, albumin%, egg shape index, and shell thickness (*p* > 0.05; Table 6)].

**Table 6:** Effect of different levels of *Dunaliella salina* on egg quality of laying hens at d 60 of the experiment.

Items	Egg quality at d 60					
	Shell %	Yolk %	Albumin %	Egg shape index (%)	Haugh unit	Shell thickness (mm)
0	12.6040	25.2740	62.1220	0.7480	78.3400	0.396
0.50	11.7820	23.6760	64.5420	0.7580	75.3540	0.388
1.00	11.7220	24.7260	63.5480	0.7560	76.5240	0.366
1.50	12.4980	23.2640	64.2400	0.7780	85.1160	0.388
SEM	0.18427	0.41857	0.48553	0.00518	1.13780	0.0059
Orthogonal contrast						
Linear	0.811	0.193	0.225	0.063	0.009	0.0389
Quadratic	0.035	0.935	0.376	0.551	0.002	0.0215

SEM: Standard Error of Means.

Linear and Quadratic effects.

### 3.2 Effect of DS on plasma biochemical and thyroid hormones of laying hens

Dietary supplementation of DS did not affect plasma total protein or its fractions (ALB, GLOB, A/G ratio, *α*-glob1, α-glob2, β glob1, β glob2, and *γ* glob) (Table 7). Including DS in laying hen diets at 500 mg/kg improved liver and kidney function by decreasing plasma ALT, AST, creatinine, urea, and uric acid (linear, *p* < 0.05; Table 8). In contrast, the 1 g/kg and 1.5 g/kg groups had increased ALT, AST, creatinine, urea, and uric acid contents compared to the control. No substantial changes were detected in thyroid hormones (T3, T4) or glucose levels after DS supplementation. Dietary inclusion of DS at 1 g/kg reduced plasma TC, LDL, and VLDL (Table 9). Plasma triglyceride (as an index of VLDA) increased (linear, *p* = 0.019; quadratic, *p* = 0.029) with dietary inclusion at 1.5 g/kg when compared to the control and other treatments.

**Table 7:** Effect of different levels of *Dunaliella salina* on protein and its fractions of laying hens.

Item	TP (g/dL)	ALB (g/dL)	GLOB (g/dL)	A/G ratio	α-glob1 (g/dL)	α-glob2 (g/dL)	β glob1 (g/dL)	β glob2 (g/dL)	γ glob (g/dL)
0	4.67	2.12	2.55	0.83	0.19	0.59	0.41	0.64	0.73
0.50	7.67	4.04	3.63	1.13	0.38	0.79	0.48	0.85	1.14
1.00	7.56	3.97	3.59	1.16	0.13	0.85	0.57	0.86	1.19
1.50	7.47	3.69	3.79	1.06	0.23	0.68	0.62	0.94	1.33
SEM	0.77	0.38	0.43	0.08	0.04	0.09	0.04	0.10	0.21
Orthogonal contrast									
Linear	0.313	0.169	0.464	0.351	0.596	0.731	0.099	0.420	0.469
Quadratic	0.393	0.150	0.685	0.270	0.447	0.397	0.899	0.800	0.810

SEM: Standard Error of Means.

Linear and Quadratic effects.

TP, total protein; ALB, albumin; GLOB, globulin.

**Table 8:** Effect of different levels of *Dunaliella salina* on liver and kidney functions, T3, T4 and glucose of laying hens.

Item	AST (U/L)	ALT (U/L)	Creatinine (mg/dL)	Urea (mg/dL)	Uric acid (mg/dL)	T3 (ng/mL)	T4	Glucose (mg/dL)
0	232.50	31.17	0.74	5.24	9.81	0.83	0.67	281.40
0.50	212.85	16.09	0.41	2.62	5.57	0.56	0.41	341.25
1.00	338.55	32.19	0.75	3.99	9.16	0.81	0.61	269.35
1.50	408.00	40.31	0.86	6.07	10.24	0.56	0.43	339.45
SEM	32.067	3.921	0.063	0.570	0.756	0.06	0.05	14.10
Orthogonal contrast								
Linear	0.003	0.019	0.027	0.045	0.002	0.189	0.140	0.269
Quadratic	0.161	0.200	0.179	0.153	0.537	0.944	0.582	0.788

SEM: Standard Error of Means.

Linear and Quadratic effects.

AST, aspartate transaminase; ALT, alanine transaminase; T3, Triiodothyronine; T4, Thyroxine.

**Table 9:** Effect of different levels of *Dunaliella salina* on total cholesterol, triglycerides, high-density lipoprotein, low-density lipoprotein, and very low-density lipoprotein of laying hens.

Item	TC (mg/dL)	TG (mg/dL)	HDL (mg/dL)	LDL (mg/dL)	VLDL (mg/dL)
0	82.11	835.50	41.46	269.00	186.05
0.50	95.89	887.05	50.02	321.50	237.81
1.00	72.11	747.55	34.23	207.50	149.51
1.50	138.70	1366.00	46.11	363.00	273.20
SEM	12.01	96.29	3.06	31.12	22.56
Orthogonal contrast					
Linear	0.163	0.019	0.944	0.554	0.346
Quadratic	0.239	0.029	0.781	0.426	0.378

SEM: Standard Error of Means.

Linear and Quadratic effects.

TC, Total cholesterol; TG, triglyceride; HDL, high-density lipoprotein; LDL, low-density lipoprotein; VLDL, very low-density lipoprotein.

### 3.3 Antioxidant’s status and immune response to DS supplementation in laying hen diets

The inclusion of DS at 1 g/kg improved the antioxidant status by numerically increasing SOD and TAC, and had an inhibitory effect (linear, *p* = 0.023; quadratic, *p* = 0.019) on MDA when compared to the control group (Table 10). Similarly, DS at 500 mg/kg improved the IgM (linear, *p* = 0.04; quadratic, *p* = 0.014) in comparison to the control group.

**Table 10:** Effect of different *Dunaliella salina* levels on antioxidant and laying hens’ immunity.

Item	SOD (U/mL)	TAC (mM/L)	MDA (nmol/mL)	IgG (ng/mL)	IgM (ng/mL)
0	58.90	0.68	7.05	518.50	87.08
0.50	62.62	0.52	6.22	718.50	112.40
1.00	72.75	0.84	5.56	397.50	97.75
1.50	30.57	0.41	13.22	229.00	57.26
SEM	7.62	0.09	1.23	77.92	8.20
Orthogonal contrast					
Linear	0.258	0.549	0.023	0.062	0.042
Quadratic	0.145	0.459	0.019	0.149	0.014

SEM: Standard Error of Means.

Linear and Quadratic effects.

SOD, superoxide dismutase; TAC, total antioxidant capacity; MDA, malondialdehyde; IgG and IgM, immunoglobulin.

### 3.4 DS biomass boosted the carotenoid content in the egg yolk

The HPLC analysis indicated that DS contains β-carotene (40 μg/g), astaxanthin (10 μg/g), zeaxanthin (20 μg/g), lutein (130 μg/g), and total carotenoids (1,100 μg/g). The HPLC carotenoid profiles of egg yolks fed progressively higher dosages of DS (0, 0.5, 1, and 1.5 g/kg diet) at three collection intervals after 15, 30, and 60 d are shown in Table 11. Except for β-carotene, increasing the content of DS raised the content of carotenoid components in the egg yolk (*p* < 0.05). In comparison to the control diet (0 g DS/kg), the highest dose of DS (1.5 g/kg) increased astaxanthin, zeaxanthin, lutein, and total carotenoids by 9.8, 50.44, 49.19, and 84.21% (*p* < 0.05), respectively, and decreased β-carotene by 38.61% (*p* < 0.05).

**Table 11:** Influence of *Dunaliella salina* supplementation levels on egg yolk carotenoid content (μg/g) in laying hens at three collecting periods (15, 30, and 60 days).

Trait	Collecting period (CP) days	DS levels (g/kg diet) (DS)				Overall mean	Significance		
		0 g	0.5 g	1.0 g	1.5 g		DS	CP	DS* CP
β-carotene	15 days	2.65 ± 0.011	1.54 ± 0.02	1.56 ± 0.011	1.35 ± 0.01	1.77^c^			
	30 days	2.65 ± 0.011	2.01 ± 0.01	2.11 ± 0.005	1.81 ± 0.02	2.15^a^			
	60 days	2.66 ± 0.011	1.49 ± 0.01	1.48 ± 0.005	1.72 ± 0.005	1.84^b^			
	Overall mean	2.66^a^	1.68^c^	1.72^b^	1.63^d^		**	**	**
Astaxanthin	15 days	3.51 ± 0.046	3.60 ± 0.02	3.59 ± 0.03	3.65 ± 0.04	3.59^b^			
	30 days	3.51 ± 0.046	3.57 ± 0.04	4.11 ± 0.05	4.33 ± 0.01	3.88^a^			
	60 days	3.52 ± 0.040	3.64 ± 0.005	3.57 ± 0.02	3.60 ± 0.02	3.58^b^			
	Overall mean	3.52^d^	3.60^c^	3.76^b^	3.86^a^		**	**	**
Zeaxanthin	15 days	4.28 ± 0.023	5.32 ± 0.01	5.91 ± 0.01	5.58 ± 0.02	5.27^b^			
	30 days	4.28 ± 0.023	5.69 ± 0.03	8.58 ± 0.03	8.38 ± 0.04	6.73^a^			
	60 days	4.28 ± 0.024	5.72 ± 0.01	5.23 ± 0.02	5.37 ± 0.01	5.15^c^			
	Overall mean	4.28^d^	5.58^c^	6.57^b^	6.44^a^		**	**	**
Lutein	15 days	7.14 ± 0.011	8.51 ± 0.01	8.90 ± 0.03	8.85 ± 0.04	8.35^b^			
	30 days	7.14 ± 0.011	8.43 ± 0.02	14.03 ± 0.05	14.49 ± 0.06	11.02^a^			
	60 days	7.15 ± 0.017	9.03 ± 0.01	8.35 ± 0.02	8.64 ± 0.01	8.29^c^			
	Overall mean	7.14^d^	8.66^c^	10.43^b^	10.66^a^		**	**	**
Total carotenoids	15 days	44.25 ± 0.13	58.10 ± 0.18	59.89 ± 0.20	59.69 ± 0.19	55.48^b^			
	30 days	44.25 ± 0.13	55.73 ± 0.23	116.06 ± 0.49	124.48 ± 0.54	85.13^a^			
	60 days	44.25 ± 0.12	60.49 ± 0.31	54.46 ± 0.25	60.39 ± 0.24	54.90^c^			
	Overall mean	44.25^d^	58.11^c^	76.80^b^	81.52^a^		**	**	**

CP, Collecting period (days); DS, *D. salina* microalgae.

^a–d^Mean within a column (having different letters) or row (having different letters) are significantly different.

*p ≤ 0.05, **p ≤ 0.01, NS = non-significant.

The egg collection period impacted the carotenoid profile of the egg yolk (*p* < 0.05). The carotenoid profile was most affected by egg collection after 30 d, with increased contents of β-carotene, astaxanthin, zeaxanthin, lutein, and total carotenoids of 20.99, 8.04, 27.75, 31.98, and 53.43%, respectively, compared to eggs collected after 15 d. The amount of β-carotene, astaxanthin, zeaxanthin, lutein, and total carotenoids in the egg yolk decreased by 14.27, 7.67, 23.51, 24.73, and 35.51%, respectively, when the collection period was extended to 60 d, as opposed to 15 d (Table 11).

### 3.5 DS boosted the long chain omega-3 fatty acid (DHA) content in the egg yolk

The GC-FID analysis indicated that the egg yolks of the control, treated hens and basal diet had six main fatty acids: palmitic acid (PA, C16:0) and stearic acid (SA, C18:0) as SFA; oleic acid (OA, C18:1, ω9) as MUFA; and linoleic acid (LA, C18:2, ω6), alpha-linolenic acid (ALA, C18:3, ω3), and docosahexaenoic acid (DHA, C22:6, ω3) as PUFA. Laying hens fed DS exhibited considerable variation in the fatty acid concentrations and lipid quality indices of egg yolk (Tables 12, 13).

**Table 12:** Influence of *Dunaliella salina* supplementation levels on major fatty acids (%) in laying hens at three collecting periods (15, 30, and 60 days).

	Trait	Collecting period (CP) days	DS levels (g/kg diet) (DS)				Overall mean	Significance		
			0 g	0.5 g	1.0 g	1.5 g		DS	CP	DS* CP
SFA	PA	15 days	25.15 ± 0.63	26.39 ± 0.64	27.33 ± 0.65	26.45 ± 0.58	26.33			
		30 days	25.21 ± 0.58	27.23 ± 0.60	27.17 ± 0.64	28.17 ± 0.64	26.94			
		60 days	25.32 ± 0.64	25.51 ± 0.59	26.18 ± 0.58	27.05 ± 0.59	26.01			
		Overall mean	25.22^b^	26.37^a^	26.89^a^	27.22^a^		*	NS	NS
	SA	15 days	6.74 ± 0.05	8.95 ± 0.12	7.89 ± 0.075	8.88 ± 0.24	8.11^b^			
		30 days	6.81 ± 0.011	8.97 ± 0.02	9.06 ± 0.09	9.05 ± 0.18	8.47^a^			
		60 days	6.95 ± 0.063	8.65 ± 0.075	8.71 ± 0.06	7.77 ± 0.07	8.02^b^			
		Overall mean	6.83^c^	8.85^a^	8.55^b^	8.56^b^		**	**	**
MUFA	OA (ω9)	15 days	45.08 ± 0.92	44.08 ± 0.70	38.81 ± 0.59	38.23 ± 0.70	41.55^a^				30 days	44.85 ± 0.69	40.27 ± 0.77	40.02 ± 0.98	40.61 ± 0.81	41.43^a^				60 days	44.11 ± 0.64	37.17 ± 0.76	38.3 ± 0.59	36.36 ± 0.53	38.98^b^				Overall mean	44.68^a^	40.50^b^	39.04^c^	38.40^c^		**	**	*
		PUFA	LA (ω6)	15 days	16.72 ± 0.28	16.17 ± 0.58	15.64 ± 0.19	15.74 ± 0.41	16.06^b^				30 days	16.89 ± 0.58	18.35 ± 0.49	17.2 ± 0.35	17.32 ± 0.30	17.44^a^			
				60 days	17.11 ± 0.35	18.36 ± 0.58	17.5 ± 0.58	16.71 ± 0.24	17.42^a^			
				Overall mean	16.9^ab^	17.62^a^	16.78^b^	16.59^b^		*	*	NS
ALA (ω3)	15 days			0.50 ± 0.005	0.33 ± 0.005	0.37 ± 0.011	0.35 ± 0.005	0.38^c^			
	30 days		0.55 ± 0.005	0.49 ± 0.011	0.49 ± 0.005	0.49 ± 0.01	0.50^a^			
	60 days		0.60 ± 0.011	0.48 ± 0.005	0.44 ± 0.005	0.43 ± 0.005	0.48^b^			
	Overall mean		0.55^a^	0.43^b^	0.43^b^	0.42^b^		**	**	**
DHA (ω3)	15 days		0.42 ± 0.005	0.86 ± 0.01	0.64 ± 0.011	0.70 ± 0.011	0.65^c^			
	30 days		0.51 ± 0.005	0.88 ± 0.011	0.79 ± 0.01	0.83 ± 0.01	0.75^a^			
	60 days		0.46 ± 0.005	0.87 ± 0.005	0.77 ± 0.005	0.70 ± 0.01	0.70^b^			
	Overall mean		0.46^c^	0.87^a^	0.73^b^	0.74^b^		**	**	**

CP, Collecting period (days); DS, *D. salina* microalgae; SFA, saturated fatty acids; MUFA, monounsaturated fatty acids; PUFA, polyunsaturated fatty acids; PA, palmitic acid; SA, stearic acid; OA, oleic acid; LA, linoleic acid; ALA, alpha-linolenic acid; DHA, docosahexaenoic acid; ω9, omega-9; ω6, omega-6; ω3, omega-3.

^a–c^Mean within a column (having different letters) or row (having different letters) are significantly different.

*p ≤ 0.05, **p ≤ 0.01, NS = non-significant.

**Table 13:** Influence of *Dunaliella salina* supplementation levels on lipid nutrition indices of egg yolks at three collecting periods (15, 30, and 60 days).

Trait	Collecting period (CP) days	DS levels (g/kg diet) (DS)			
		0 g	0.5 g	1.0 g	1.5 g
MUFA/ PUFA	15 days	2.56	2.60	2.33	2.28
	30 days	2.50	2.04	2.17	2.18
	60 days	2.43	1.89	2.04	2.04
PUFA/ SFA	15 days	0.55	0.49	0.47	0.47
	30 days	0.56	0.54	0.51	0.50
	60 days	0.56	0.57	0.53	0.51
IA	15 days	0.51	0.57	0.64	0.65
	30 days	0.51	0.61	0.62	0.63
	60 days	0.52	0.61	0.62	0.65
IT	15 days	0.95	1.04	1.17	1.18
	30 days	0.94	1.09	1.12	1.14
	60 days	0.95	1.08	1.11	1.17
h/H	15 days	2.49	2.37	2.03	2.08
	30 days	2.49	2.20	2.15	2.10
	60 days	2.46	2.23	2.18	2.00

PUFA, polyunsaturated fatty acids; MUFA, monounsaturated fatty acids; SFA, saturated fatty acids; IA, the atherogenicity index; IT, the thrombogenicity index; h/H, the hypocholesterolemic/hypercholesterolemic index.

The concentrations of saturated fatty acids (PA and SA) in egg yolks increased when laying hens were fed DS. Compared to the non-supplemented group, feeding a 1.5 g DS/kg diet for 30 d increased PA by 11.74% and SA by 32.89%. Feeding DS to laying hens negatively influenced the omega-9 (OA) content in egg yolks. The OA content was reduced by 17.57% after 60 d on a 1.5 g DS/kg diet compared to the control. Feeding a 0.5 g DS/kg diet for 15 d led to a 104.76% increase in the concentration of long-chain omega-3 (DHA) and a 34% decrease in the concentration of short-chain omega-3 (ALA) in the yolk, compared to the control. The lipid nutrition indices of egg yolks, which are related to fatty acid constituents, were positively influenced by feeding DS to laying hens. These indices included the MUFA/PUFA and PUFA/SFA, IA, IT, h/H. Lipid nutrition indices of egg yolks were 1.89 in 0.5 g DS/kg diet/60 d to 2.60 in 0.5 g DS/kg diet/15 d group for MUFA/PUFA, 0.47 in 1 g DS/kg diet/15 d to 0.57 in 0.5 g DS/kg diet/60 d group for PUFA/SFA, 0.51 in control/15 and 30 d groups to 0.65 in 1.5 g DS/kg diet/15 and 60 d groups for IA, 0.94 in 0.5 g DS/kg diet/30 d group to 1.18 in 1.5 g DS/kg diet/15 d group for IT, and 2.00 in 1.5 g DS/kg diet/60 d group to 2.49 in 0.5 g DS/kg diet/15 and 30 d groups for h/H (Table 13). The DS and basal diets also showed adequate lipid nutrition indices (Table 14).

**Table 14:** Major fatty acids and lipid nutrition indices of *D. salina* microalgae and basal diet of laying hens.

	SFA		MUFA	PUFA			Lipid nutrition indices				
	PA	SA	OA (ω9)	LA (ω6)	ALA (ω3)	DHA (ω3)	MUFA/ PUFA	PUFA/ SFA	IA	IT	h/H
DS	26.31	2.08	4.22	2.31	4.34	0	0.63	0.22	2.82	1.69	0.41
Diet	13.83	2.39	32.66	47.56	1.64	0.02	0.66	3.03	0.20	0.36	5.92

DS, *D. salina* microalgae; SFA, saturated fatty acids; MUFA, monounsaturated fatty acids; PUFA, polyunsaturated fatty acids; PA, palmitic acid; SA, stearic acid; OA, oleic acid; LA, linoleic acid; ALA, alpha-linolenic acid; DHA, docosahexaenoic acid; ω9, omega-9; ω6, omega-6; ω3, omega-3; IA, the atherogenicity index; IT, the thrombogenicity index; h/H, the hypocholesterolemic/hypocholesterolemic index.

## 4 Discussion

Natural carotenoids, which are essential nutritive, medicinal, and potent antioxidant phytochemicals, such as β-carotene, astaxanthin, zeaxanthin, and lutein, are extensively synthesized in DS microalgae (41). Research on the dietary use of DS in laying hens is relatively limited, whereas there are numerous studies examining the dietary effects of DS on the growth performance of broiler chickens. In the current study, the most important observation was that dietary DS had a more pronounced effect on the deposition of carotenoids, other pigments, and the lipid profile of egg yolk than on blood parameters. The results showed that hen egg production and feed conversion were substantially improved by 1 g DS supplementation. DS in the diet improved FCR (42). The high β-carotene levels in DS may account for the observed improvements in EP and FCR in the present study. These were absorbed into the intestine and subsequently transported to the liver and tissues via portomicrons, and to the egg yolk by lipoproteins modified for deposition in the yolk (43). *Dunaliella salina* stands out among microalgae due to its ability to accumulate high levels of β-carotene (44). As a potent antioxidant, β-carotene safeguards cellular components like lipids, proteins, and DNA from oxidative damage. This powerful antioxidant plays crucial roles in enhancing various physiological functions, including immune system function, adipocyte function, reproduction, and gene expression (45). Since animals cannot synthesize β-carotene, dietary supplementation offers a promising strategy for bolstering immunity (46). Furthermore, previous research has shown that β-carotene can modulate the gut microbiota, potentially enhancing intestinal mucosal health in chicks (47). Inclusion of DS in laying hen diets at 500 mg/kg improved the immune response, liver and kidney function, and reduced MDA under heat stress conditions in the present study. Recent research has demonstrated that DS exhibits both anti-inflammatory and antioxidant properties owing to its high levels of total carotenoids, particularly β-carotene, and unsaturated fatty acids, including alpha-linolenic acid (48). Therefore, the hepatoprotective effects of DS are evident in improvements in serum biomarkers and liver histology (49).

The limited bioavailability of carotenoids in humans has prompted researchers to investigate whether microalgal carotenoids are also bioavailable to layer hens. Because of the carotenoid metabolism in laying hens, more nutritious eggs are produced to satisfy the demands of the consumer market (21). In the present study, we produced carotenoid-enriched eggs by gradually adding microalgal carotenoids from DS to the diets of laying hens. The addition of increasingly higher doses of dietary DS in the current study was positively correlated with the concentrations of astaxanthin, zeaxanthin, lutein, and total carotenoids in the egg yolk (*p* < 0.05). Similar to our findings, Fernandes et al. (42) augmented the concentration of total carotenoids in egg yolk with DS. A similar trend was observed for astaxanthin and total carotenoids in the egg yolk of layer hens fed progressively higher doses of the microalgae, *Haematococcus pluvialis* (50). The concentration of total carotenoids, β-carotene, lutein, and zeaxanthin in egg yolk increased with the addition of increasing doses of microalgae, *Nannochloropsis oculate* (36), *Sargassum dentifebium* (51), and Chlorella (52) in the diet of the laying hens. As increasing dosages of DS were added to the laying hen diet, the amount of β-carotene in egg yolk decreased and was the lowest of all the carotenoids in the current study compared to the highest one, lutein. These findings confirm prior results that lutein content is highest and β-carotene content is lower in egg yolks than other carotenoids (36, 52, 53).

The xanthophylls, astaxanthin, zeaxanthin, and lutein, are not provitamin A carotene, but β-carotene is well-known as a provitamin. Laying hens can effectively convert β-carotene into vitamin A, which might explain why egg yolks contain less β-carotene than other xanthophylls (54, 55). In the current study, the maximum carotenoid content in the egg yolk was found when the eggs were collected after 30 d; however, when the collection period was extended to 60 d, the carotenoid content began to decrease. Extending the feeding of supplementary DS to over 30 d in the current study may have contributed to this decline by prompting an adverse mechanism that limited the ability of chickens to absorb and deposit microalgal carotenoids in the yolk. Magnuson et al. (50) observed a similar trend when they extended the collecting period from 3 w to 6 weeks, where total carotenoid and astaxanthin contents in the egg yolk of layer hens fed 80 mg *Haematococcus pluvialis*/kg diet fell from 114 and 36.2 mg/kg to 86.1 and 31.1 mg/kg, respectively. The astaxanthin content in egg yolks of laying hens that were fed a diet supplemented with varying concentrations of palm toco concentrate and algae biomass containing astaxanthin for 8 weeks reached its highest concentration on day 10. After that, the levels gradually declined over time. The transfer efficiency of astaxanthin ranged from 7.6 to 14.9% (56). In contrast, Fernandes et al. (42) demonstrated that the total carotenoid content in egg yolks of laying hens fed DS biomass gradually increased until the end of the experiment at week 52.

The current findings indicate that laying hens can absorb and deposit the supplemented DS carotenoids (β-carotene, astaxanthin, zeaxanthin, lutein, and total carotenoids) in their egg yolks. There have been numerous reports on the transmission of natural carotenoids, particularly those from microalgae, in layer hen egg yolks (22, 35, 36, 42, 50, 54). Carotenoids and other lipid phytochemicals are absorbed in the same manner as vitamin E in the small intestine via chylomicrons and then transferred via the lymphatic system to the bloodstream, where they are stored in the liver (50, 57). In the liver, hepatocytes release modified lipoproteins for deposition in the egg yolk, which delivers carotenoids to the egg yolk (35, 58). High quantities of antioxidant carotenoids in egg yolk, particularly the xanthophylls, astaxanthin, zeaxanthin, and lutein, protect the fatty acids inside the yolk from oxidation and boost the egg’s shelf life and health benefits (42, 50). Enriching eggs with natural carotenoids, particularly the xanthophylls astaxanthin, zeaxanthin, and lutein, may increase the bioavailability of carotenoids in humans and increase the health benefits of carotenoid-fortified eggs.

Omega-3 PUFAs, such as ALA (C18:3), eicosapentaenoic acid (EPA, C20:5), and DHA (C22:6) may prevent and control various cardiovascular and neurological problems in addition to immunological difficulties (59). Recently, consumer interest in foods enriched with n-3 PUFAs has increased. Consequently, researchers are becoming increasingly interested in enriching eggs with omega-3 PUFAs, particularly DHA, to meet consumer preferences for healthier and more nutritious eggs. In the present study, we fed DS microalgal biomass to laying hens to enrich their eggs with omega-3 PUFAs. The fatty acid composition of the yolk was affected by adding DS to the diet at 0.5, 1, and 1.5 g/kg, particularly when DS was added to the diet for up to 15 d, which caused the optimization of yolk PUFAs, particularly the long chain PUFA (DHA, ω3). These findings align with prior study results reporting that adding microalgal biomass to hen diets might improve the levels of omega-3 PUFAs, particularly DHA, in egg yolk. Previous studies have shown that this is possible with *Nannochloropsis limnetica* (22, 26), *Staurosira* sp.*, Desmodesmus* sp.*, Nannochloropsis oceanica* (60), *Chlorella fuscaas*, *Phaeodactylum tricornutum, Isochrysis galbana, Nannochloropsis oculata* (54), and *Nannochloropsis oculate* (36). Although the DS lipid profile alone contains short-chain omega fatty acids, such as OA, LA, and ALA (41), feeding DS to laying hens in this study decreased the amount of these unsaturated fatty acids, while increasing the amount of DHA, a long-chain PUFA, in the egg yolks. These findings suggest that laying hens fed DS are encouraged to convert OA, LA, and ALA into DHA before deposition in the yolk. Numerous investigations (22, 24, 26, 36, 54, 60) have confirmed these results and reported that egg yolks of hens fed a diet high in C-18 unsaturated fatty acids contain low C-18 unsaturated fatty acids and high DHA contents.

According to the current study, DS administration to laying hens affected the lipid nutrition indices of egg yolks. DS improved the MUFA/PUFA, particularly at 0.5 g/kg, improving the oxidative stability of egg yolks owing to the high proportion of MUFAs, which is in agreement with the findings of Wołoszyn et al. (40). Similarly, it improved the PUFA/SFA to higher than 0.45, particularly at 0.5 g/kg, making the egg yolks able to improve heart health and retard the progress of cardiovascular disorders. Foods with PUFA/SFA < 0.45 have been reported to be unsuitable and unsafe for human nutrition because they increase blood cholesterol levels (61, 63). Furthermore, by feeding DS to the laying hen in this investigation, the egg yolks retained their low IT, low IA, and high h/H. These favorable indices are consistent with earlier recommendations (62) and demonstrate the beneficial effects of egg yolks in preserving cardiovascular function.

Overall, the high DHA content of egg yolks and their positive nutritional indices make eggs a promising functional, healthy food with great potential for controlling blood lipid profiles.

## 5 Conclusion

Feeding DS microalgae to heat-stressed laying hens enhanced the nutritional quality of their eggs, egg production, and immune response. The highest dose of DS (1.5 g/kg diet) substantially increased astaxanthin, zeaxanthin, lutein, and total carotenoid contents in egg yolks. The low dosage of DS (0.5 g/kg diet) resulted in eggs with a high DHA content and favorable nutritional indices. Feeding DS to laying hens resulted in eggs with high levels of bioactive carotenoids and beneficial omega-3 fatty acids (DHA), making these eggs a prospective functional health food.

## Data Availability

The original contributions presented in the study are included in the article/supplementary material, further inquiries can be directed to the corresponding author.
